# Supplementary material for: Intermetallic PdZn nanoparticles catalyze the continuous-flow hydrogenation of alkynols to cis-enols
Source: Commun Chem. 2021 Dec 13;4:175. doi: 10.1038/s42004-021-00612-0 (PMC9814770; doi:10.1038/s42004-021-00612-0)
Supplement: Supplementary file 1 — Supplementary Information [file 42004_2021_612_MOESM1_ESM.pdf]

Supporting Information

Intermetallic PdZn nanoparticles catalyze the continuous-flow hydrogenation of alkynols to *cis*-enols

Xiao Chen [1†], Chuang Shi [1†], Xing-Bao Wang [2,3†], Wen-Ying Li [2,3]*, Changhai Liang [1]*

[1] State Key Laboratory of Fine Chemicals, Laboratory of Advanced Materials and Catalytic Engineering, School of Chemical Engineering, Dalian University of Technology, Dalian 116024, China.

[2] State Key Laboratory of Clean and Efficient Coal Utilization, Taiyuan University of Technology, Taiyuan 030024, China.

[3] Key Laboratory of Coal Science and Technology (Taiyuan University of Technology), Ministry of Education, Taiyuan 030024, China.

* Correspondence should be addressed to Wen-Ying Li; ying@tyut.edu.cn and Changhai Liang; changhai@dlut.edu.cn

† These authors contributed equally to this work.

Table of contents

1. Materials……………………………………………………………………….…..3
2. Adsorption survey of ZnO……………………………………………….…....…...3
3. Computational models and methods……………………………………….……4
4. Hydrogenation of BYD in the batch reactor……………………………………….5
5. Additional characterization information and catalytic results……………………..……6
Supplementary Figure 1…………………………………………………….……..6
Supplementary Figure 2…………………………………………………..………..7
Supplementary Figure 3…………………………………………………….……..8
Supplementary Figure 4…………………………………………………..………..9
Supplementary Figure 5………………………………………………………………..10
Supplementary Figure 6………………………………………………………………..11
Supplementary Figure 7………………………………………………………………..12
Supplementary Figure 8………………………………………………………………..13
Supplementary Figure 9………………………………………………………………..14
Supplementary Figure 10……………………………………………………...…15
Supplementary Figure 11………………………………………………………16
Supplementary Figure 12………………………………………………………17
Supplementary Figure 13………………………………………………………18
Supplementary Figure 14……………………………………………………...……19
Supplementary Figure 15………………………………………………………20
Supplementary Figure 16………………………………………………………21
Supplementary Figure 17………………………………………………………22
Supplementary Scheme 1………………………………………………………23
Supplementary Table 1………………………………………………..……..24
Supplementary References……………………………………………..……25

Supplementary Methods

1. Materials

All raw materials and solutions for preparation were acquired from commercial corporations and could be used without further disposed. High-purity nano-zinc oxide (ZnO, Aladdin, >98%) as a high PZC support was used to adsorb anion precursors. Sodium-tetrachloropalladium (Na_2PdCl_4, Aladdin, >98%) was used as metallic Pd precursor. BYD (>98%) was purchased from Macklin Inc. Propargyl alcohol (>99%) was purchased from Alke reagent company. 2-Methyl-3-butyne-2-ol (>98%), 3-hexyn-1-ol (>97%), 3-phenyl-2-propyn-1-ol (>96%), Pd/SiO_2, Pd/C, Pd/TiO_2, $Pd/CaCO_3$, $PdAg/Al_2O_3$ catalysts, and Lindlar catalyst (5 wt.% $Pd/CaCO_3$ poisoned with lead) were purchased from Aladdin reagent Co. Ltd..

2. Adsorption survey of ZnO

In the EpHL experiments, dry ZnO was weighed out to achieve the SL of 2000 $m^2\ L^{-1}$ for 50 mL of solution according to the given SL calculation formula in order to obtain the best SL and dosed into 100 mL flasks. Strong base and strong acid solutions were used at different pH values in the scope from 1 to 13 using HCl and NaOH. Surface loading calculation formula is as follow,

$$SL(m^2\ L^{-1}) = \frac{SA_{support}\ (m^2\ g^{-1})\ * m_{support}(g)}{V_{DIwater}(L)} \qquad (1)$$

where $SA_{support}$ attributed to the surface area of ZnO, $m_{support}$ attributed to the mass of ZnO, and $V_{DIwater}$ attributed to the volume of water.

Aqueous solutions of 3.0 mmol L^{-1} Na_2PdCl_4 (PdTc) were prepared and poured in 100 mL beakers. $NH_3{\cdot}H_2O$ and HNO_3 were used to change the initial pH of the PdTc aqueous solutions from 0.4 to 11.0 ZnO was weighed out to acquire the required 1000 $m^2\ L^{-1}$ SL in 100

mL of aqueous solution, and to permit a high enough PdTc concentration for simple and accurate ICP measurement. The ZnO powder was dosed into 100 mL of various pH previously adjusted solution and put on the magnetic stirrers for 2 h. After stirring, the final pH values were measured by the pH meter and 4 mL of well-mixed supernatant liquor was filtered for ICP analysis. Initial solutions at every pH value without contacting ZnO supports, were also measured with ICP to obtain initial precious metal concentrations. Adsorption data and the initial pH value are drawing versus the final pH value. Surface density Γ calculation formula is as follow:

$$\Gamma(\mu\text{mol m}^{-2})=\frac{\Delta c_{Pd}(\text{mg L}^{-1})*10^{6}(\mu\text{mol mol}^{-1})}{SL(\text{m}^{2}\text{ L}^{-1})*M_{Pd}(\text{g mol}^{-1})*1000(\text{mg g}^{-1})} \qquad (2)$$

Where Δc_{Pd} attributes to the differential concentration of Pd and M_{Pd} denotes the Pd relative atomic mass.

3. Computational models and methods

The Pd(111) and PdZn(111) surfaces were modeled on *p*(6×6) supercells involving four layered slabs with the vacuum layer height of 15 Å. Herein, the two layers at the bottom of surface were fixed into their bulk positions and the top layer was allowed to relax. The results discussed in this study were obtained using the Doml^3 package [1]. Considering the exchange-correlation functional, the generalized gradient approximation (**GGA**) with the Perdew-Burke-Ernzerhof function (**PBE**) was selected [2]. The double-numeric polarized (**DNP**) basis set was chosen, and the DFT semi-core pseudopotential (**DSSP**) was adopted which includes the norm-conserving pseudopotential and the relativistic effect to treat the core electrons. To guarantee the cutoff radius was larger enough, the value was set of 4.5 Å. Geometric optimization was firstly carried out to obtain the adsorption structure with the minimum total energy. The convergence criteria of geometric optimization were set as 1×10^{-5} Ha for total

energy, 0.004 Ha Å^{-1} for maximum force of every atom, and 0.005 Å for maximum displacement. The electronic self-consistent field (**SCF**) tolerance was set at 10^{-5} Ha. A 3×3×1 Monkhorst-Pack grid k-point was set in geometric optimization with smearing of 0.001 eV.

The adsorption energies (E_{ads}) was defined by

$$\Delta E_{ads} = E_{(adsorbate/surface)} - E_{(adsorbate)} - E_{(bare\ surface)} \qquad (3)$$

where $E_{(adsorbate/surface)}$ is the total energy of an adsorbate bound to the Pd or PdZn slab, $E_{(adsorbate)}$ is the total energy of an isolated molecule or intermediate, $E_{(bare\ surface)}$ is the total energy of the bare slab. The reaction energy (ΔE_{rxn}) and the activation energy (E_a) were calculated using following equations:

$$\Delta E_{rxn} = E_{product} - E_{reactant} \qquad (4)$$

$$E_a = E_{transition} - E_{reactant} \qquad (5)$$

where $E_{product}$, $E_{reactant}$, and $E_{transition}$ are the total energies of product, reactant, and transition state, respectively.

4. Hydrogenation of BYD in the batch reactor

Liquid-phase hydrogenation of BYD was carried out in a 50 mL stainless autoclave containing 0.1 g as-prepared Pd/ZnO catalyst, 25 mL of feedstock with 5 wt.% BYD, 1 wt.% 1,2-propylene glycol (as internal standard) and 96% water (as solvent) and 2.0 MPa H_2 at 80 °C. The reaction system was stirred vigorously (700 rpm) to eliminate the diffusion effect. Liquid aliquots were taken periodically using a dip tube. The products were analyzed by gas chromatography (Agilent 7890B-GC).

5. Additional characterization information and catalytic results

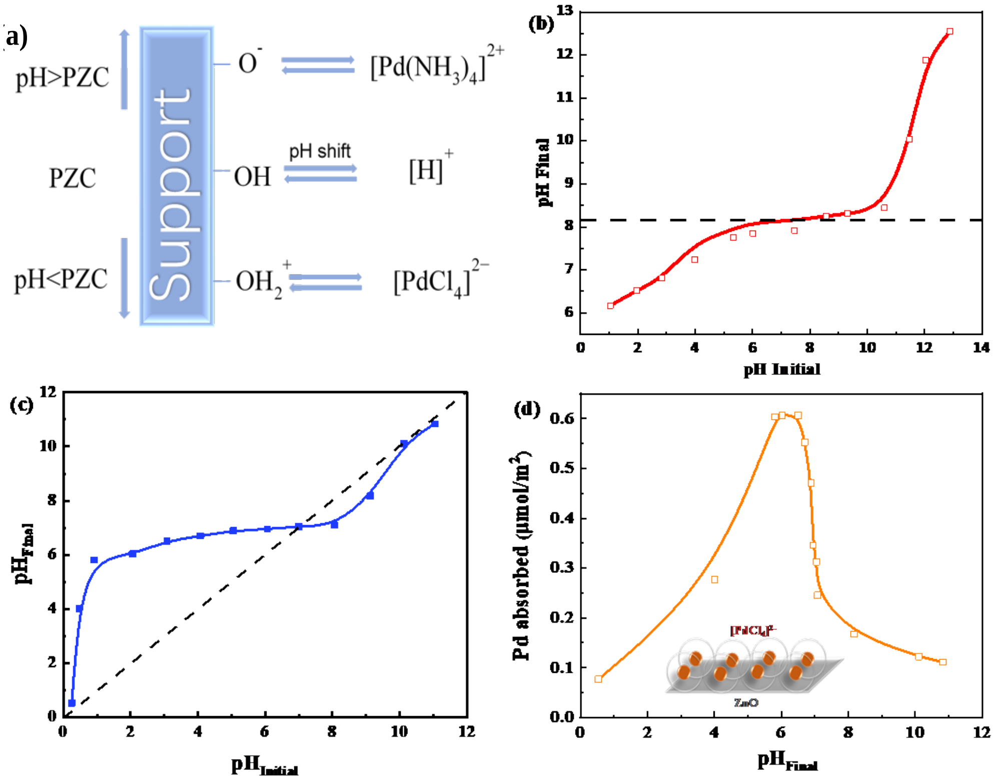

Supplementary Figure 1. (a) The mechanism of electrostatic adsorption. (b) pH shift data for metal-free solutions contacted with ZnO at 2000 m^2 L^{-1}. (c) pH shift of impregnation solution and (d) metal uptake survey of $[PdCl_4]^{2-}$ on ZnO.

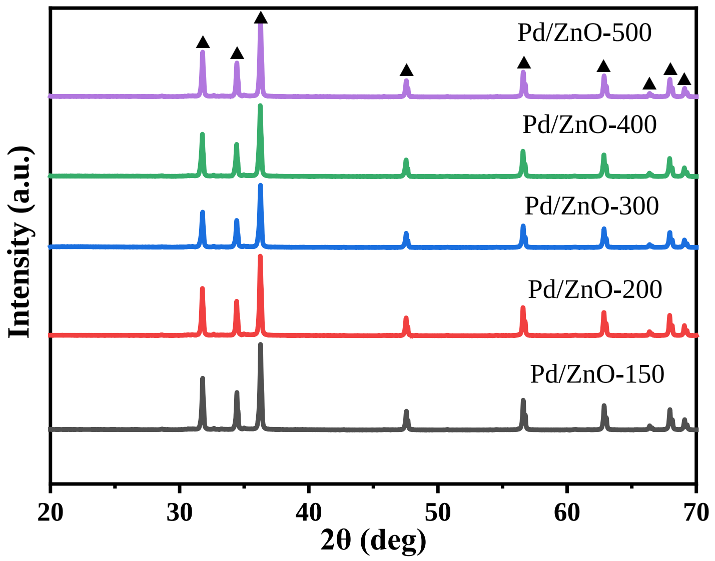

Supplementary Figure 2. XRD patterns of Pd/ZnO-*T* samples.

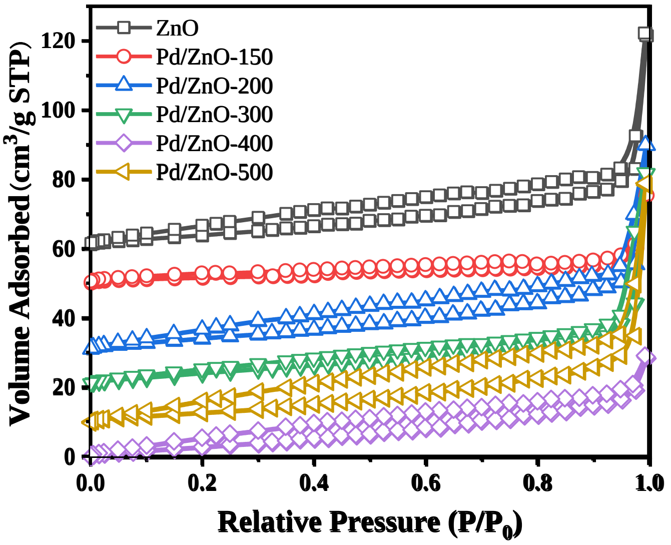

Supplementary Figure 3. N_2 adsorption/desorption isotherms at -196 °C of the ZnO and Pd/ZnO-*T* samples.

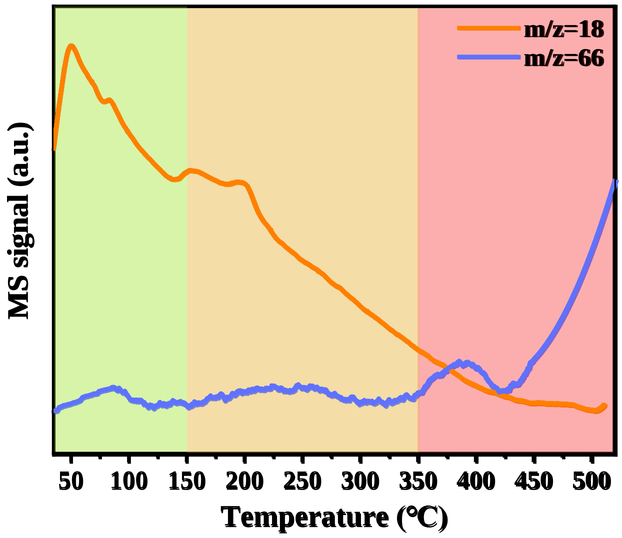

Supplementary Figure 4. Mass spectrum of H_2O and ZnH_x for the Pd/ZnO sample under 5 vol.% H_2/Ar from RT to 520 °C with 5 °C min^{-1}.

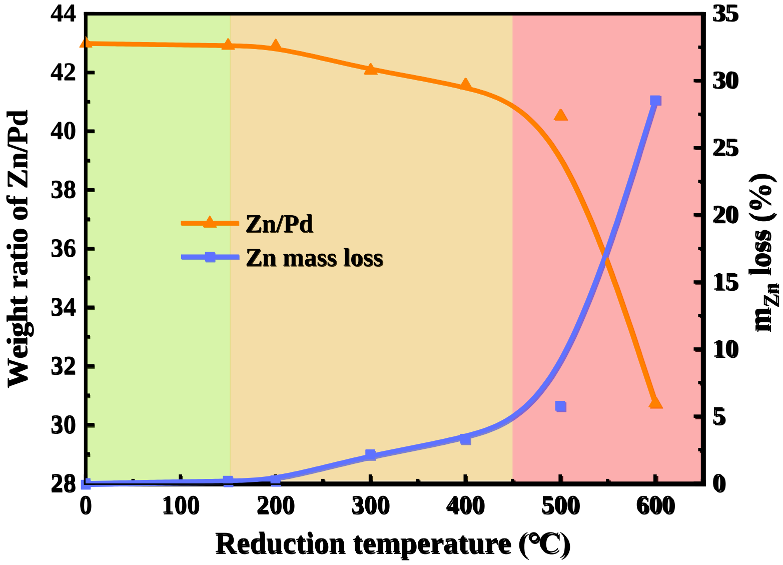

Supplementary Figure 5. ICP-AES measurement of Pd/ZnO-*T* samples.

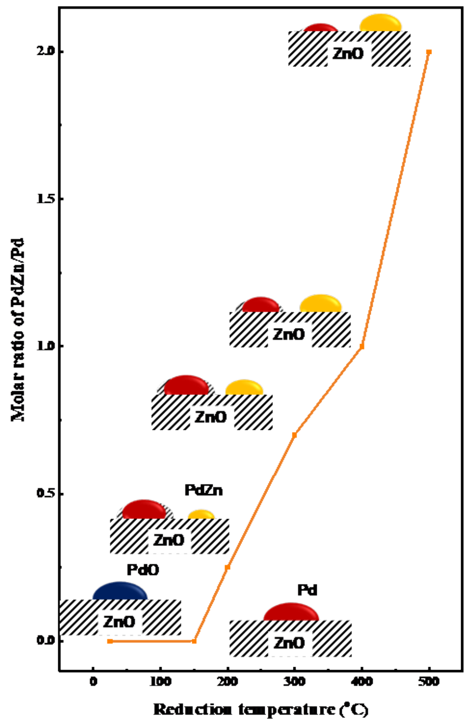

Supplementary Figure 6. The molar ratio of PdZn/Pd versus the reduction temperature (PdO: navy blue, Pd: wine red, PdZn: yellow).

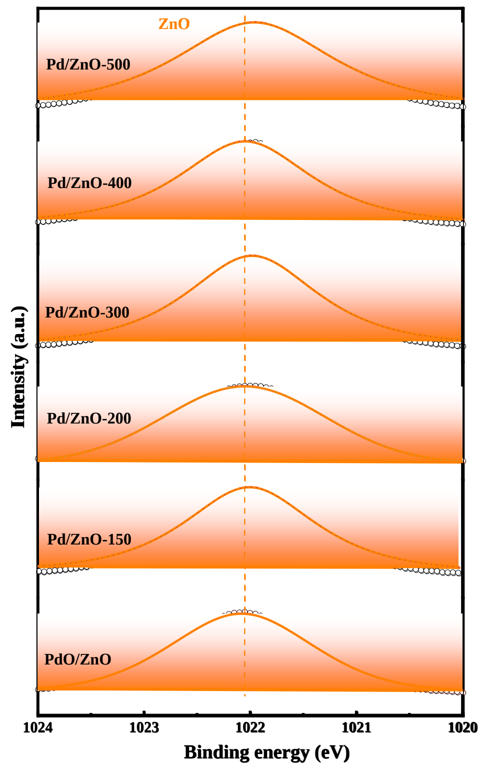

Supplementary Figure 7. XPS of Zn 2*p* region of unreduced PdO/ZnO and Pd/ZnO-*T* samples.

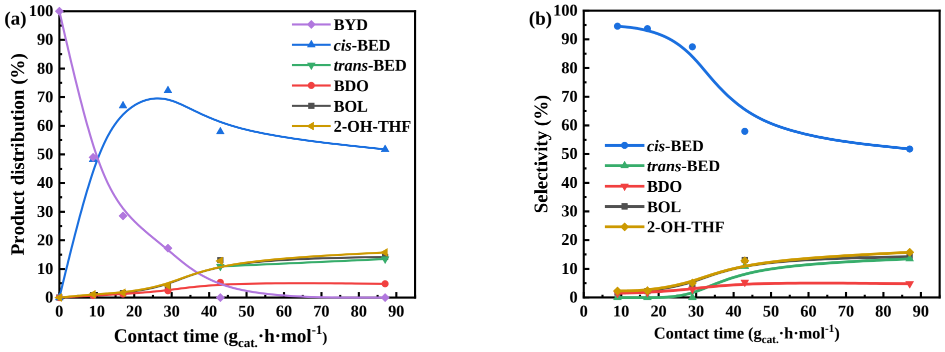

Supplementary Figure 8. (a) Product distribution and (b) selectivity of the BYD hydrogenation versus the contact time at 2 MPa H_2 and 80 ℃ over Pd/ZnO-150 catalyst.

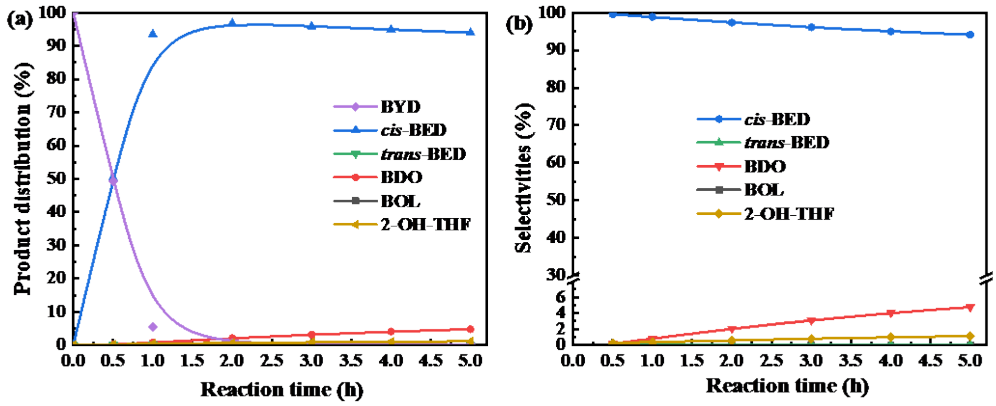

Supplementary Figure 9. (a) The variation of the relative concentration and (b) the products selectivities with reaction time for the hydrogenation of BYD over Pd/ZnO-400 catalyst in a batch reactor versus time (Reaction conditions: 0.1 g catalyst, reaction temperature 80 °C, 2 MPa H_2, and 25 g 5 wt.% BYD aqueous solution).

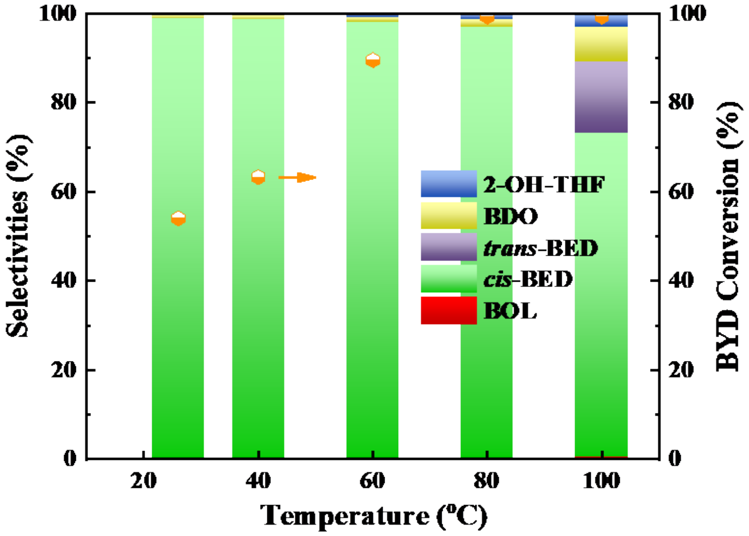

Supplementary Figure 10. The effect of reaction temperature on the selective hydrogenation of BYD over Pd/ZnO-400 catalyst (Reaction conditions: 0.1 g catalyst, 2 MPa H_2, contact time 29 $g_{cat.}\cdot h\cdot mol^{-1}$, and the volume ratio of H_2 to liquid feed 600).

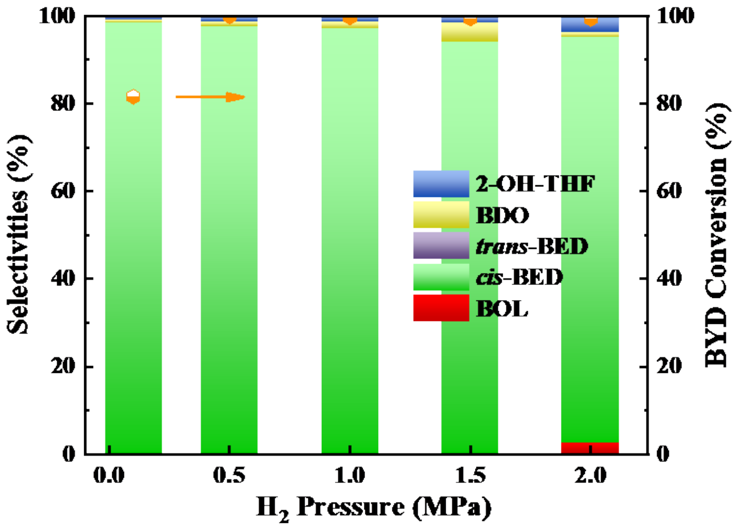

Supplementary Figure 11. The effect of H_2 pressure on the selective hydrogenation of BYD over Pd/ZnO-400 catalyst (Reaction conditions: 0.1 g catalyst, reaction temperature 80 ^oC, contact time 29 $g_{cat.}{\cdot}h{\cdot}mol^{-1}$, and the volume ratio of H_2 to liquid feed 600).

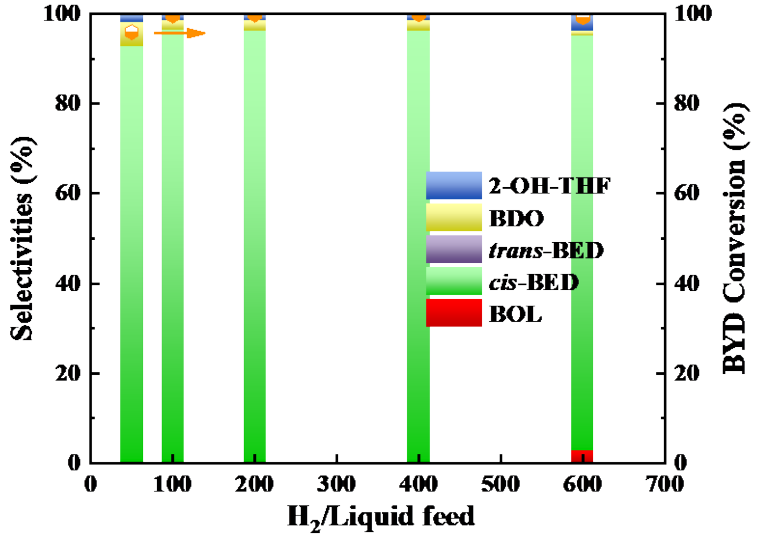

Supplementary Figure 12. The effect of the volume ratio of H_2 to liquid feed on the selective hydrogenation of BYD over Pd/ZnO-400 catalyst (Reaction conditions: 0.1 g catalyst, reaction temperature 80 ^{o}C, 2 MPa H_2, and contact time 29 $g_{cat.}$·h·mol^{-1}).

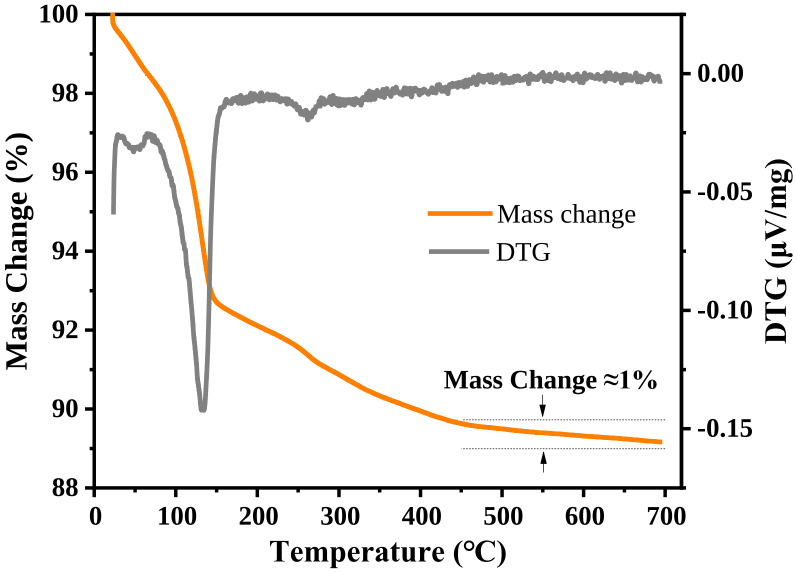

Supplementary Figure 13. TG/DTG measurement of the spent Pd/ZnO-400 catalyst after the stability testing in 5% O_2/Ar up to 700 °C with 5°C min^{-1}.

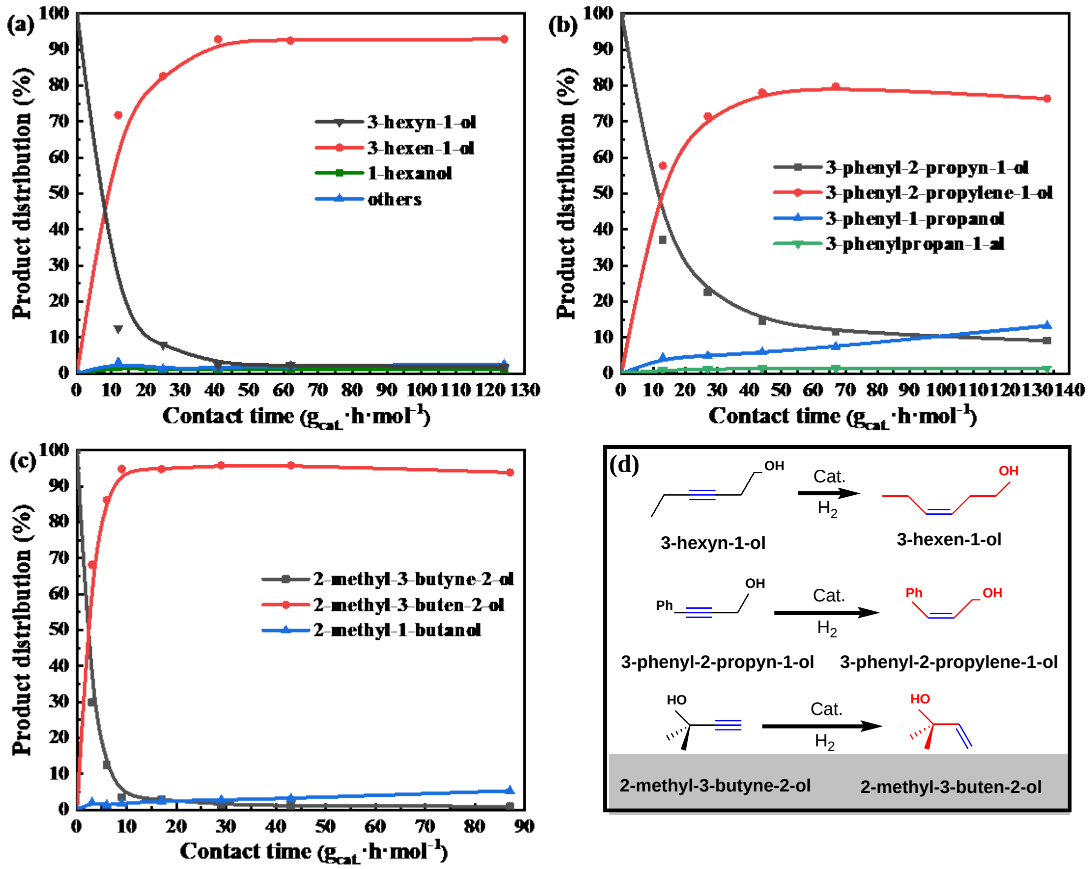

Supplementary Figure 14. Product distributions of the hydrogenation of (a) 5 wt.% 3-hexyn-1-ol in methanol at 130 °C and 3 MPa, (b) 3 wt.% 3-phenyl-2-propyn-1-ol in methanol at 100 °C and 3 MPa, and (c) 5 wt.% 2-methyl-3-butyne-2-ol in water at 50 °C and 1 MPa versus the contact time over Pd/ZnO-400 catalyst. (d) The reaction pathways of hydrogenation of alkynols.

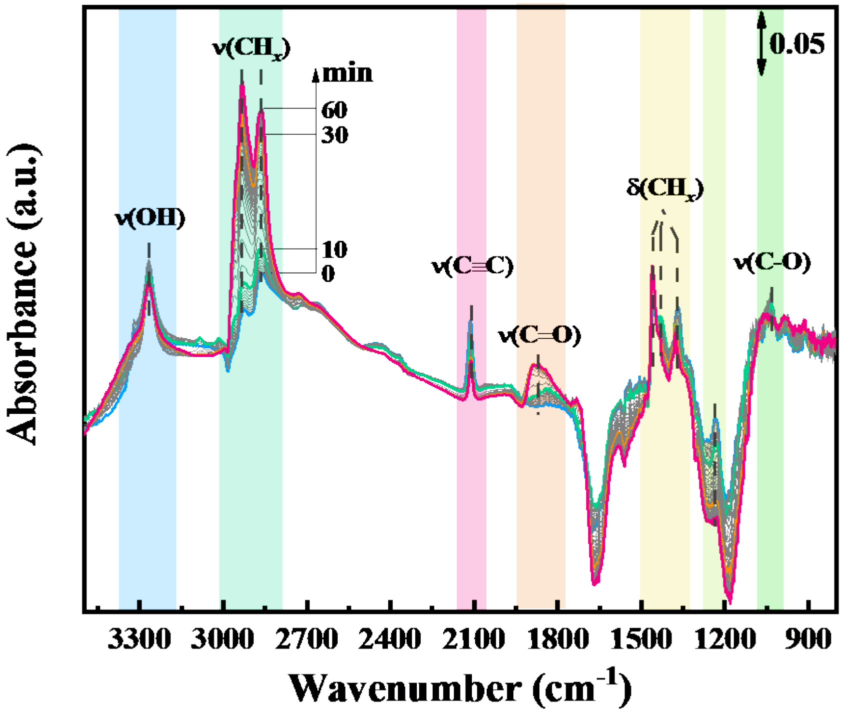

Supplementary Figure 15. *In situ* DRIFT spectra during the propargyl alcohol hydrogenation over the Pd/ZnO-150 catalyst at 30 ^{o}C.

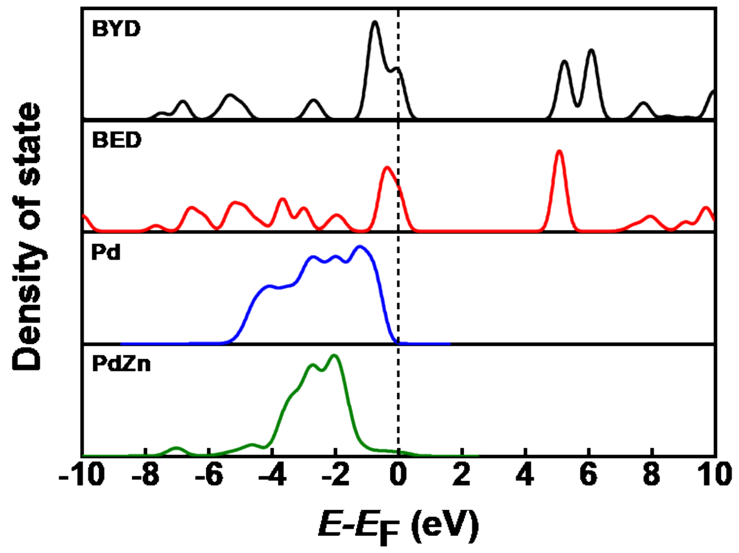

Supplementary Figure 16. The calculated total electronic density of states of BYD, BED, Pd, and PdZn.

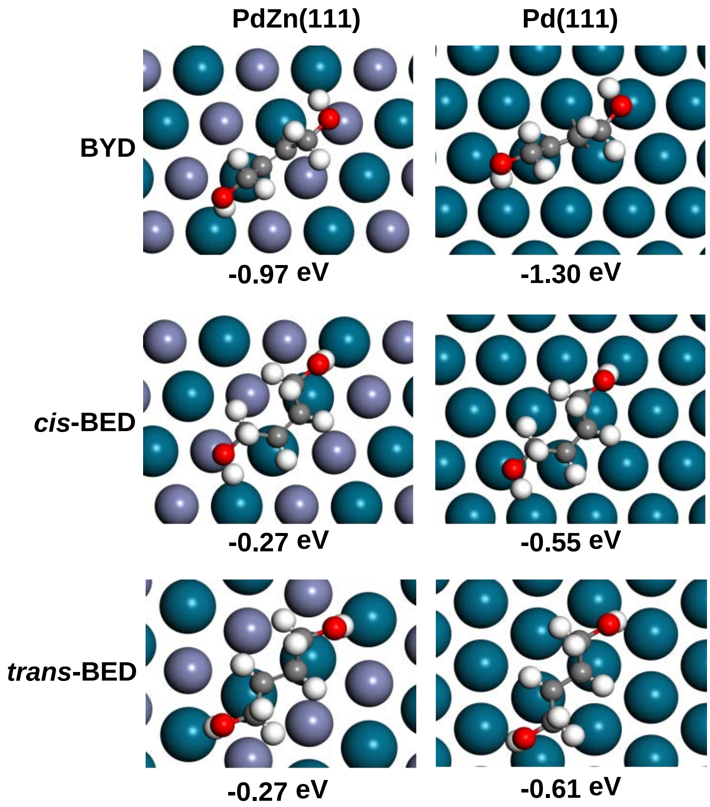

Supplementary Figure 17. Adsorption configurations and energies of BYD, *cis*-BED, and *trans*-BED on PdZn (111) and Pd (111), respectively.

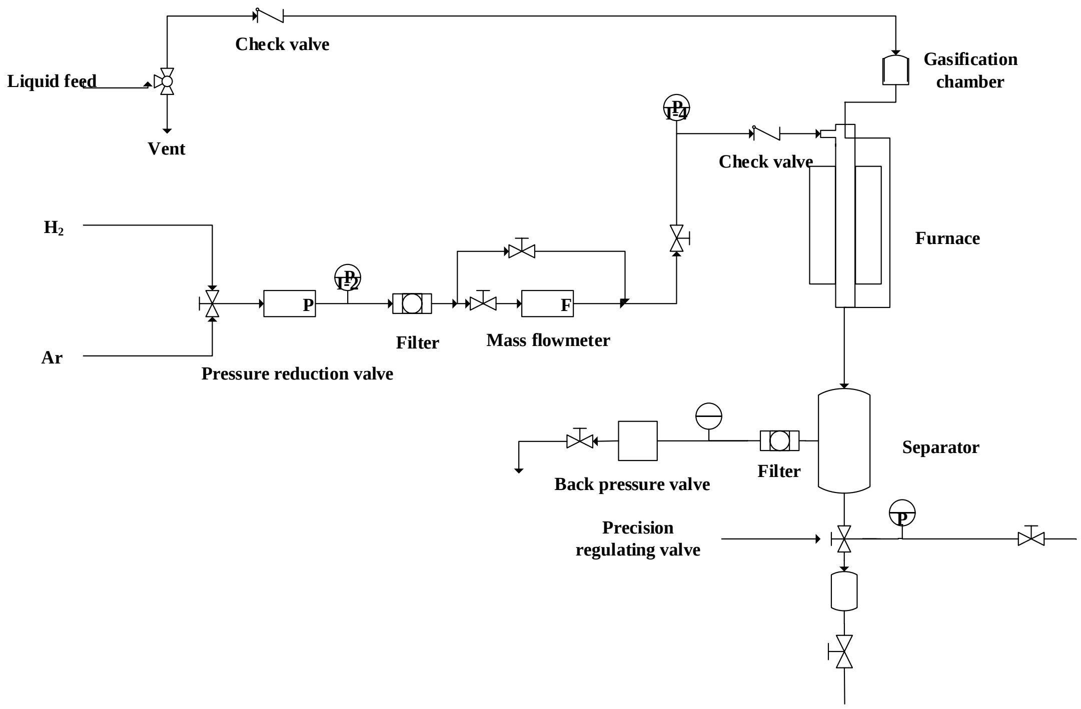

Supplementary Scheme 1. The unit of high pressure fixed bed continuous flow stainless steel catalytic reactor.

Supplementary Table 1. Composition and physicochemical properties of Pd/ZnO-*T* catalysts

Sample	Metal loading (wt.%)	S_{BET} ($m^2\ g^{-1}$)	V_p ($cm^3\ g^{-1}$)	D_p (nm)	CO uptake (μmol g^{-1})	D_{Pd} (%)	TOF (min^{-1})
ZnO	-	18	0.03	3.9	-		
Pd/ZnO-150	0.8	7	0.04	3.3	6.9	9	90
Pd/ZnO-200	0.8	16	0.09	2.9	20.6	27	23
Pd/ZnO-300	0.8	17	0.04	5.1	15.5	20	30
Pd/ZnO-400	0.8	13	0.03	2.9	8.4	11	71
Pd/ZnO-500	0.9	12	0.11	3.1	18.9	22	35

Supplementary References

[1] Delley, B. From molecules to solids with the DMol^3 approach. *J. chem. Phys.* **113**, 7756−7764 (2000).

[2] Perdew, J. P., Burke, K. & Ernzerhof, M. Generalized gradient approximation made simple. *Phys. Rev. Lett.* **77** (3865–3868) 1996.
